# Impact of social determinants on COVID-19 infections: a comprehensive study from Saudi Arabia governorates

**DOI:** 10.1057/s41599-022-01208-2

**Published:** 2022-10-07

**Authors:** Abdallah S. A. Yaseen

**Affiliations:** National Centre for Social and Criminological Research, Giza, Egypt

**Keywords:** Health humanities, Medical humanities

## Abstract

The last two years have been marked by the emergence of Coronavirus. The pandemic has spread in most countries, causing substantial changes all over the world. Many studies sought to analyze phenomena related to the pandemic from different perspectives. This study analyzes data from the governorates of the Kingdom of Saudi Arabia (the KSA), proposing a broad analysis that addresses three different research objectives. The first is to identify the main factors affecting the variations between KSA governorates in the cumulative number of COVID-19 infections. The study uses principal component regression. Results highlight the significant positive effects of the number of schools in each governorate, and classroom density within each school on the number of infections in the KSA. The second aim of this study is to use the number of COVID-19 infections, in addition to its significant predictors, to classify KSA governorates using the *K*-mean cluster method. Findings show that all KSA governorates can be grouped into two clusters. The first cluster includes 31 governorates that can be considered at greater risk of Covid infections as they have higher values in all the significant determinants of Covid infections. The last objective is to compare between traditional statistical methods and artificial intelligence techniques in predicting the future number of COVID-19 infections, with the aim of determining the method that provides the highest accuracy. Results also show that multilayer perceptron neural network outperforms others in forecasting the future number of COVID-19. Finally, the future number of infections for each cluster is predicted using multilayer perceptron neural network method.

## Introduction

**Fig. 1:**
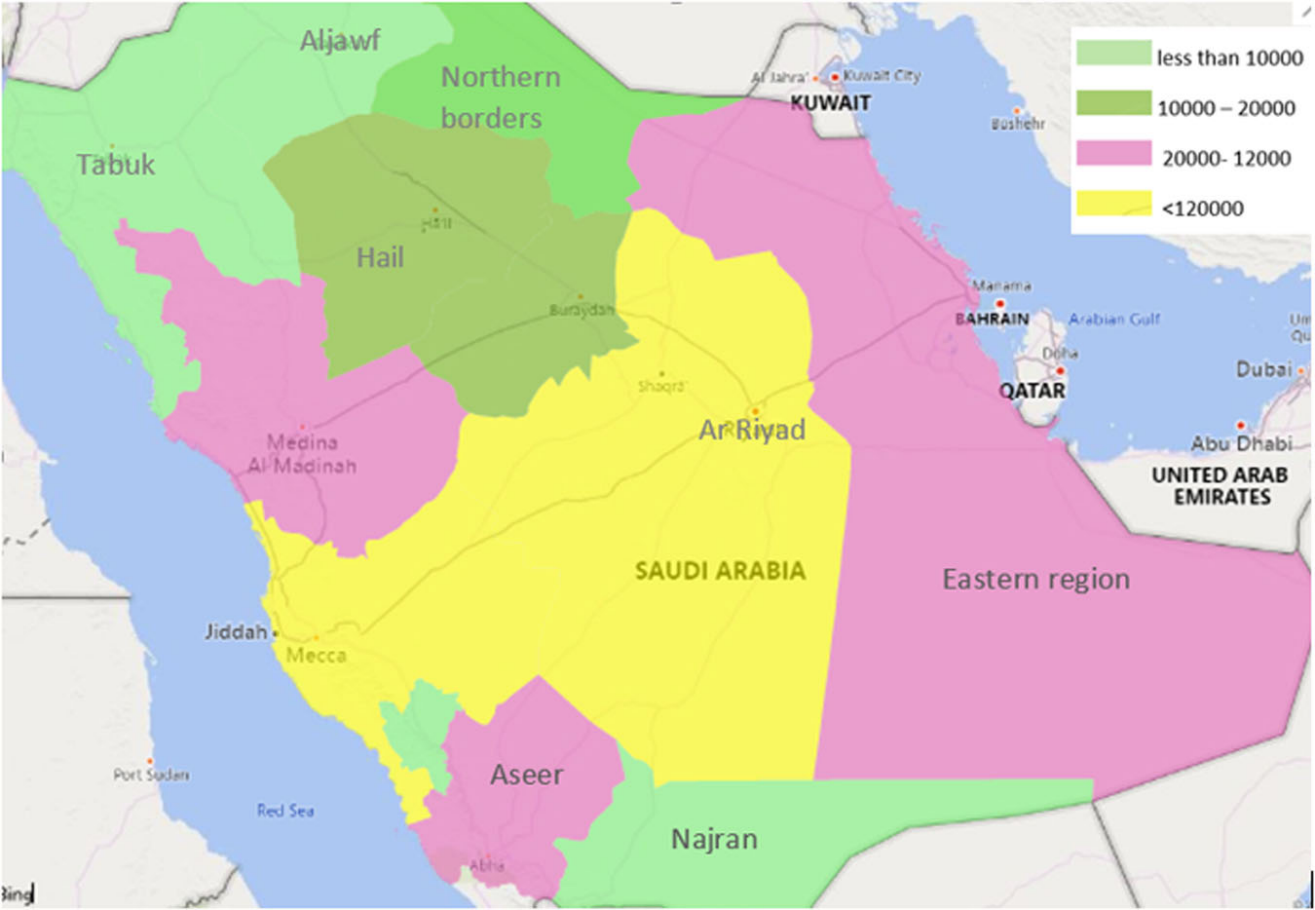
KSA regions classified by number of COVID-19 infections.

COVID-19 is a recently discovered infectious disease that was first discovered in Wuhan, China and ravaged the world to become a pandemic. By January 2021, World Health Organization (WHO) announced more than 93 million infected cases of COVID-19 and more than 2 million deaths around the world (World Health, 2020). The first case of COVID-19 in the Kingdom of Saudi Arabia (KSA) was reported on March 2, 2020. By July 2021, according to the Ministry of Health-KSA, the epidemic has spread in 206 cities and yields more than 400 thousand infected cases which caused more than 6000 deaths in the KSA. Figure 1 presents the 13 KSA regions with respect to the COVID-19 cases. It is clear that most of the infections concentrate in the middle of KSA, more specifically in Ar Ryiad and Mecca regions.

KSA started applying early preventive measures to prevent the spread of COVID-19. To help stopping its spread globally and locally, KSA followed some strictest approaches. Some of these approaches are closing the international borders, two grand mosques in Mecca and Medina for any tourists, air travel, all public offices, and all educational organizations.

Analyzing the widespread of COVID-19 is of great importance, and should be seriously investigated to control the outbreak. Statistical techniques, and soft computing methods play a prominent role in studying and investigating the pandemic, and the different aspects related to it. The key questions that COVID-19 raises pertain to the reasons behind the decrease or increase in the number of infections, and to the expected scenarios the world will face. The study by Alkhowailed et al. (2020) tackles the effect of meteorological parameters, such as temperature, humidity, and wind speed on the spread of the ongoing COVID-19 in Saudi Arabia. A similar study is conducted in China by Xie and Zhu (2020). Romero Starke et al. (2020) investigate the effect of age on the propensity of infection using meta regression analysis. Eybpoosh et al. (2021) investigate the severity and mortality of COVID-19 infection in HIV-infected patients using regression analysis. The same investigation is conducted for individuals with mental illness‏ in Chang et al. (2020). Xiong et al. (2020) analyze the difference in infection rate in population subgroups across combinations of demographic characteristics, using logistic regression model.

Moreover, various studies aim to build forecasting methods to anticipate the expected number of infections and deaths due to COVID-19. Such techniques are diverse, and include a huge number of prediction methods. However, two main approaches receive a special interest, which are the autoregressive integrated moving average (ARIMA)-based models, and the artificial neural network (ANN) methods. For instance, Elsheikh et al. (2021b) employ deep learning methods to anticipate the total numbers of confirmed cases, recovered cases, and deaths in the KSA, using the long short-term memory (LSTM) network. Several articles apply different variants of ARIMA models on daily infections/deaths of COVID-19 to predict incidence of COVID-19. These studies include Benvenuto et al. (2020), Kufel (2020), Sahai et al. (2020), Perone (2020), Sharma et al. (2020), and Alabdulrazzaq et al. (2021). In contrast, other studies tackle the same problem using various types of ANN networks, such as the acritical intelligence-based methods. These studies include Pal et al. (2020), Wieczorek et al. (2020), Namasudra et al. (2021), Dhamodharavadhani et al. (2020), Huang et al. (2020), Dandekar and Barbastathis (2020), Tamang et al. (2020), Distante et al. (2020), Jena et al. (2021), and Mollalo et al. (2020).

The present study aims to conduct a broad statistical, machine learning and deep learning analysis, with three main objectives.:

1. The first is to investigate the possible determinants of the increase/decrease in the cumulative number of COVID-19 infections in KSA governorates. Studying Covid infections in KSA governorates from a social perspective was not tackled before.
2. The second is to incorporate the number and determinants of COVID-19 infections in a cluster analysis, in order to classify KSA governorates into homogeneous groups or clusters. This can provide more information about which governorates highly suffer from Covid pandemic and how to efficiently deal with it in the light of infections’ determinants.
3. The third is to forecast the future daily number of infections in each constructed cluster to anticipate the future number of infections in KSA governorates.

To the best of our knowledge, connecting these three objectives that entail regression modeling, classification technique and time series forecasting methods in one study narrating the current story of Covid-19 and anticipating its future have not been accomplished before.

The article is organized in five main sections in addition to the conclusion. The section “Data sources” presents the data sources of this study. An overview for KSA governorates is provided in the section “Overview of KSA governorates”. Various statistical methods used in this study are highlighted in the section “Data analysis methods” and subsections embedded in. Finally, the results are explained and investigated in the section “Results”. The study ends with some conclusion comments. The attached Appendix contains auxiliary tables and figures.

## Data sources

Data were collected from two different resources. Daily and cumulative numbers of infected cases in Saudi Arabia governorates are collected from KAPSARC data portal^1^ during the period from March 22, 2020 to July 11, 2021. The cumulative number of infected cases in KSA governorates represents the total number of infected cases for 135 governorate on July 11, 2021. The characteristics of the different KSA governorates used in this study are obtained from the Saudi General Authority for Statistics, which offers detailed data about all KSA governorates and regions in the services statistical bulletin. All data used in this study are collected on July 11, 2021. Table 1 presents the main characteristics of the gathered data for all governorates. Data of COVID-19 infections collected from KAPSARC data portal are attached to their matching data from the KSA services statistical bulletin, and all incorporated in one data model, using the Power BI software, employed for data transformation and preparations.

**Table 1:** Descriptive statistics of the study variables.

Variable	Scale	Code/Label	Mean	Standard deviation	Minimum	Maximum
Population size	Governorate	POPULATION	196,364	572,128	6798	5,236,901
Number of houses	Governorate	HOUSES	255,471	263,336	3019	909,228
Number of undergraduate male schools	Governorate	MSCHOOLS	84	106	8	859
Number of undergraduate female schools	Governorate	FSCHOOLS	87	110	3	874
Average of classroom intensity in male schools	Governorate	MAV INTENSITY	25.23	6.68	12.73	42.73
Average of classroom intensity in female schools	Governorate	FAV INTENSITY	24.20	7.19	10.10	43.47
Number of employees	region	EMPLOYEES	744,361	1,110,039	64,847	3,887,768
Percentage of people 65+ years old	region	OLD	7.06	5.99	1.30	24.50
Average of monthly salary for employees	region	AV SALARY	14,849	2967	12,421	21,189
Percentage of people covered by health insurance	region	INSURANCE	27.61	10.50	16.50	49.24
Number of hospitals	region	HOSPITALS	3	7	1	54
Number of beds	region	BEDS	511	1479	50	14,110
Number of nurses	region	NURSES	935	3087	25	30,719
Number of doctors	region	DOCTORS	384	1283	18	12,386
Number of healthy centers	region	CENTERS	149	110	43	415

## Overview of KSA governorates

The Kingdom of Saudi Arabia is broadly classified to 13 regions, which is the broadest classification. Each region is classified into a number of governorates, in addition to the head of region called “Emarah.” The number of governorates in each region ranges from to 3 to 23 governorates. The total number of governorates is 137. Governorates are further subdivided into sub-governorates known as “Markez.” The largest region, according to population size and number of governorates, is “Ar Riyad,” which includes the capital of the KSA with the same name. The second largest region is “Mecca,” which includes 16 governorates. Table 2 presents study variables means in each region. It can be noted that “Ar Riyad” and “Mecca” regions have the highest values in most of the variables which is expected for the largest regions. Tabuk, Hail, Al Bahah, Al Jawf, Jazan, Najran and North region have relatively lower values. Other regions have moderate values in the study variables.

**Table 2:** Means of the study variables in KSA regions.

•  indicates rank of region value is >80% of all regions.  indicates rank of region value is >60% of all regions.  indicates rank of region value is >40% of all regions.  indicates rank of region value is >20% of all regions.  indicates rank of region value is <80% of all regions.

• *OLD, INSURANCE and AV SALARY in each region are estimated by the average of all governorates in that region.

## Data analysis methods

The analysis in this study is divided into three connected parts. First, the differences between KSA governorates are investigated with respect to the cumulative number of infections. Afterwards, KSA governorates are clustered using the cumulative number of infected cases, and the significant variables proven to be related to it, using the *K*-mean cluster method, which is a machine learning technique. In the final part, four forecasting approaches are compared to determine the best model to fit the daily number of infected cases for each cluster. Recommendations are proposed in light of the study results presented in the “Conclusion”.

Since the study has different goals, various statistical and data analysis software are incorporated. Power BI is used for incorporating and preparing all data in one data model. SPSS v.26 is used for building elementary regression model, clustering data and creating forecasting models using seasonal exponential smoothing (SES), seasonal autoregressive-integrated moving average (SARIMA) models and multilayer perceptron (MLP) neural network. R package V4.1.2 is invoked to test and deal with heteroscedasticity problem cursed the model-dependent variable. NCSS v.2021 is used for estimating the parameters of the principal component regression model. Finally, MATLAB v.2019 is employed for constructing long short-term memory (LSTM) neural network models.

For the possible determinants of COVID-19-infected cases, the study depends on the services statistical bulletin published by the Saudi General Authority for Statistics, which provides regional-based as well as governorate-based statistics. The study also includes all the variables that may be relevant to COVID-19 infections. This results in three sets of explanatory variables in the proposed model. Since population size and density are the strongest factors affecting the cumulative number of infected cases in Saudi governorates, the first group of predictors includes population size, and number of houses. The second group of predictors involves the demographic variables available in a governorate base, including the number and average classroom density of the undergraduate schools for boys, and the number and average classroom density of undergraduate schools for girls. The third group contains variables that are not available in a governorate base, yet available in regional base instead, whence governorates in the same region have the same values. These variables pertain to the health sector, and other demographic variables, as presented in Table 1.

### Regression analysis

Regression models are defined as statistical techniques aimed to find the relationships between one or more dependent variables using a set of explanatory variables. The traditional expression for regression model is

$$Y = XB + \varepsilon$$

where *Y* in the left-hand side represents the outcome while *X* represents a set of explanatory variables possibly affecting *Y* using a set of model coefficients *B* and *ε* is the error term of the model.

The primary goal of the regression analysis is to explain the difference in the cumulative number of infected cases between KSA governorates. Whence we attempt to build a regression model to find the determinants of the increases/decrease in the cumulative number of infected cases. The dependent variable is therefore the cumulative number of infected cases for all governorates.

### Regression model setup

First, the data for regression analysis are prepared by applying the logarithm transformation for the dependent and independent variables, in order to reduce heterogeneity and outliers, except for OLD, which has no outliers. Next, the predictors of the model are determined. Choosing the appropriate explanatory variables is a state of art. Due to the great number of covariates, a flexible practical approach is followed, starting with the construction of simple linear regressions, where each covariate is separately entered to the model. Only the significant variables in the aforementioned models are then included in one model to measure the conditional effect of each predictor. Thirteen variables show significant effect in the simple regression models, so all of them are included in the proposed model.

Nevertheless, estimating the conditional effect of the predictors is considered an obstacle in the proposed regression model, taking into account the incident correlation between the predictors. For the regression model used in this study, most of the variables are highly correlated with each other, particularly with population size and number of houses, leading to severe multicollinearity problem. Pearson correlation coefficients matrix of the explanatory variables in Table (A-1) in the Appendix concludes that there is a high correlation between variables. Thereby, it is challenging to estimate the effects of the variables correlated with population size, and number of houses, while keeping population size and number of houses in the same model.

### Regression model diagnoses

In order to diagnose model performance, an elementary model is estimated using the ordinary least-squares (OLS) estimation method, and all the predictors to calculate model residuals. Collinearity diagnoses measures are also computed using eigenvalues and conditional number. As shown in Table (A-2) in the Appendix, most of the predictors are collinear with each other. Another problem that violates regression assumptions arises by plotting studentized residuals versus fitted values (Blatná, 2006). Figure (A-1) in the Appendix clearly reveals a heteroscedasticity problem. Besides, two outlier points are detected which represent Ar Riyad and Gedda governorates. However, comparing results with and without the two outliers shows no substantial difference.

Since we have multiple linear regression assumptions violated, Box–Cox transformation and principal component regression are employed instead of the logarithm transformation for the model outcome and ordinary least-square regression technique. Box–Cox transformation is frequently employed to circumvent the violation of normality or homogeneity assumption. The general form of Box–Cox transformation is

$$x\left( \lambda \right) = \left\{ {\begin{array}{*{20}{c}} \displaystyle{\frac{{x^\lambda - 1}}{\lambda },\,\lambda \,\ne\, 0} \\ {\ln \left( x \right),\,\lambda = 0} \end{array}} \right.,$$

where *x*(*λ*) is the transformed variable after applying Box–Cox transformation that depends on the value of *λ*. Choosing different values for *λ*, leads to different variants of Box-Cox transformation. As *λ* tends to zero, Box–Cox transformation is equivalent to the logarithm transformation.

Using R package “caret”, the number of infections is transformed by estimating *λ* with −0.2 which is its maximum-likelihood estimator. The assumption of Homoscedasticity is then tested using studentized Breusch–Pagan test (Breusch and Pagan, 1979) and single global test (Peña and Slate, 2006). Single global test can be adopted to check various linear regression assumptions such as linear relationship, skewness, kurtosis, and heteroskedasticity. Furthermore, Durbin–Watson test is performed to validate the assumption of no correlation between residuals. Table (A-3) in the Appendix presents tests statistics and p-values for single global test and studentized Breusch–Pagan test which both confirm homoscedasticity of the residuals after applying Box–Cox transformation. Analogically, Durbin–Watson test assures there is no autocorrelation between residuals.

To address the multicollinearity problem, principal component regression is employed instead of the OLS regression model. The motivation behind utilizing the principal component regression is to use the eigenvectors of the scaled and centered predictors, instead of the predictors themselves, after excluding the eigenvectors with low variance; i.e., small eigenvalues. Since eigenvectors are orthogonal, the model overcomes the collinearity problem. Afterwards, the eigenvectors are re-transformed to the original predictors for the purpose of obtaining interpretable estimates and calculating their significance levels. Regression results are presented in the sub-section “Regression model results”.

### Cluster analysis

The following section examines the results of the estimated regression model by employing the cumulative number of infected cases and its significant determinants, in order to cluster KSA governorates into groups of similar units, using *K*-mean cluster method. The steps of performing *K*-mean cluster are straightforward:

1. First *k* data items are arbitrarily chosen from the dataset as initial cluster centroid.
2. Second each data item is assigned to the cluster to which object is most similar that is determined based on the Euclidean distance between each item and the cluster mean.
3. Afterwards the mean of each cluster is re-calculated and updated.
4. The algorithm iterates between the second and third step until convergence.

However, the *K*-mean cluster method has one drawback which is the unavailability of any selection criterion for the number of clusters *k*. Thus, the number of clusters is determined by two criteria. The first is using hierarchal clusters, calculating Wald linkage coefficients, and applying elbow rule using the scree diagram chart (Multivariate Solutions, 2014). The second is by conducting Silhouette analysis for finding the optimal number of clusters. Cluster analysis is implemented, and its results are discussed in the sub-section “Cluster analysis results”.

### Forecasting analysis

This part aims to incorporate the composed clusters in forecasting models, so as to predict the daily number of COVID-19 cases in each cluster. Forecasting methods can generally be classified into conventional statistical techniques, and new artificial intelligence methods. In this study, four approaches are included in a comparison to find the best model to fit the daily number of infected cases for each of the clusters, which can be used for future predictions.

ARIMA models, also known as the Box–Jenkins models, first proposed by Box et al. (2015) are basically linear estimators regressed on past values (the autoregressive terms) or past prediction errors (the moving average terms). Traditional ARIMA models are denoted as ARIMA(*p*,*q*,*d*) where *p*,*q*,*d* are parameters of the autoregressive model order, the degree of differencing and moving average model order, respectively. Seasonal ARIMA or SARIMA models are modified versions of the traditional ARIMA models, adapted for seasonal data. Seasonality is a regular pattern of change that repeats over *S* time periods, where *S* is the number of time periods until the pattern is repeated again. SARIMA models have the form of SARIMA(*p*,*d*,*q*)_*x*_(*P*,*D*,*Q*)*S*. For more details see de Oliveira and Oliveira (2018).

Furthermore, the exponential smoothing (ES) methods refer to the traditional procedures that continually revise the forecast in light of recent information about the estimated data by assigning exponentially decreasing weights as observation gets dated. A special type of the exponential smoothing models is the seasonal exponential smoothing (SES) models, which include seasonal term in the model. Several approaches to the exponential smoothing techniques are presented; among which the Holt–Winters additive/multiplicative models are the most common (de Oliveira and Oliveira, 2018).

In contrast to the above statistical methods that can easily be represented in simple equations explicitly defining independent and dependent variables, ANN methods come with the terminology of inputs and outputs linked through layers of neutrons that resemble the biological nervous systems. Two common classes of ANN are typically used for time series forecasting: the multilayer perception (MLP) and the long short-term (LSTM) networks. MLP networks belong to feed-forward artificial neural networks, which typically involve three types of layers, input layer, hidden layer (*s*), and output layer. LSTM networks are evolved versions of recurrent neural networks that use feedback connection to make it more complex than traditional MLP networks. However, such complexity comes with the merit of solving the complicated problems that MLP networks fail to resolve (Elsheikh et al., 2021a).

The structure of a typical LSTM neural is composed of cells. The output of each cell is the result of multiple processes. LSTM networks store relevant past information in an additional memory called cell state. The information in cell states is controlled by gates. Each cell has three gates; input gate, forget gate, and output gate. Data can be removed from or added to the cell state using activation gates that apply sigmoid activation functions to data.

## Results

The following section presents the results of the three main parts of the analysis: i.e., principal regression analysis, cluster analysis, and forecasting analysis, respectively.

### Regression model results

We run principal component regression to obtain unbiased estimates in the existence of colinear predictors. The estimated model parameters can be shown below in Table 3.

**Table 3:** Estimated principal component regression coefficients of the study regression model.

Variable	Estimated coefficient	Standardized coefficient	*T*-statistic	*P*-value
*l.POPULATION*	0.16***	0.13	9.12	<0.00001
*l.HOUSES*	0.19***	0.15	2.45	0.02
*l.MSCHOOLS*	0.19***	0.12	3.68	0.0009
*l.FSCHOOLS*	0.18***	0.12	3.40	0.0002
*l.MAV INTENSITY*	0.65***	0.13	2.43	0.02
*l.FAV INTENSITY*	0.48***	0.10	2.84	0.008
*l.EMPLOYEES*	−0.02	−0.02	−0.57	0.58
*OLD*	0.01	0.02	0.80	0.43
*l.INSURANCE*	−0.58***	−0.14	−2.47	0.019
*l.HOSPITALS*	0.14***	0.09	2.86	0.007
*l.BEDS*	0.11***	0.10	4.11	0.0003
*l.NURSES*	0.09***	0.10	4.81	0.00004
*l.DOCTORS*	0.10***	0.10	5.11	0.00002

***Significant at 99% confidence interval.

The results of the model show that both population size and number of houses have positive effects on the cumulative number of infected cases. Larger governorates tend to have larger cumulative number of infected cases. A significant result is the positive estimated coefficients of *l*.*MSCHOOLS*, *l*.*FSCHOOLS*, *l*.*MAV INTENSITY*, and *l*.*FAV INTENSITY*, which represent the conditional effects of the underling predictors excluding the effect of population size and density. Hence comparing governorates with similar population size and density reveals governorates with higher classroom density (for either male or female students), and/or higher number of schools (for either male or female students) tend to have higher cumulative number of infected cases compared to other governorates. Higher classroom density reflects that many students are frequently in close contact. Higher numbers of schools indicate that more students and teachers regularly leave their houses making them susceptible to contact with infected patients, which may result in their infection.

Furthermore, it is interesting to show that the percentage of individuals covered with health insurance *l*.*INSURANCE* negatively affects the cumulative number of infected cases, whereas health-related variables such as *l*.*HOSPITALS*, *l*.*BEDS*, *l*.*NURSES* and *l*.*DOCTORS* have positive coefficients. This can be interpreted as KSA government assigns more medical aids, such as hospitals, beds, nurses, and doctors, to governorates that highly suffer from COVID-19, reflecting good measures in dealing with the pandemic. The negative estimated slope of *l*.*INSURANCE* is a good indication that health insurance, with its associated care offered to citizens, decreases the number of COVID-19 infections. Finally, both *l.EMPLOYEES* and *OLD* do not seem to have a significant conditional effect. For *l.EMPLOYEES*, the insignificant effect can be due to the precautionary measures adopted in workplaces, which contribute to restricting infection among adult employees. The insignificance of *OLD* suggests that senior citizens are prone to infection as younger citizens, and that both are in equal risk, which is observed when analyzing infections of different variants of COVID-19 (Table 3).

### Cluster analysis results

**Fig. 2:**
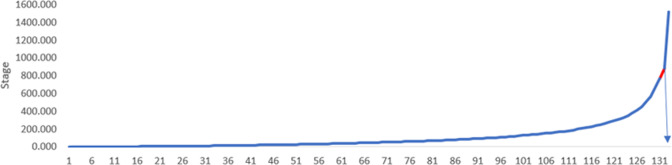
Scree diagram chart for defining number of clusters using elbow rule.

**Fig. 3:**
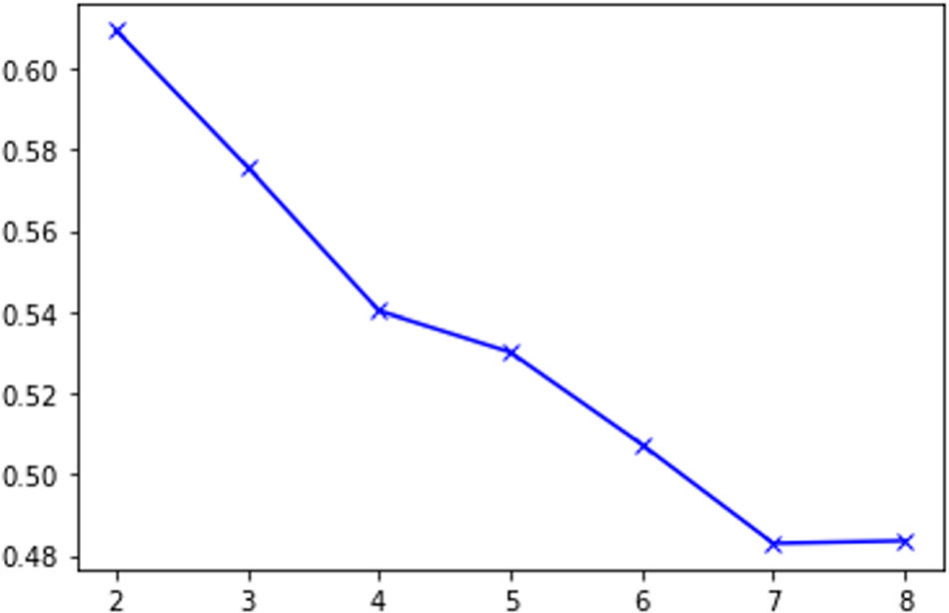
Silhouette analysis for optimal number of clusters.

**Fig. 4:**
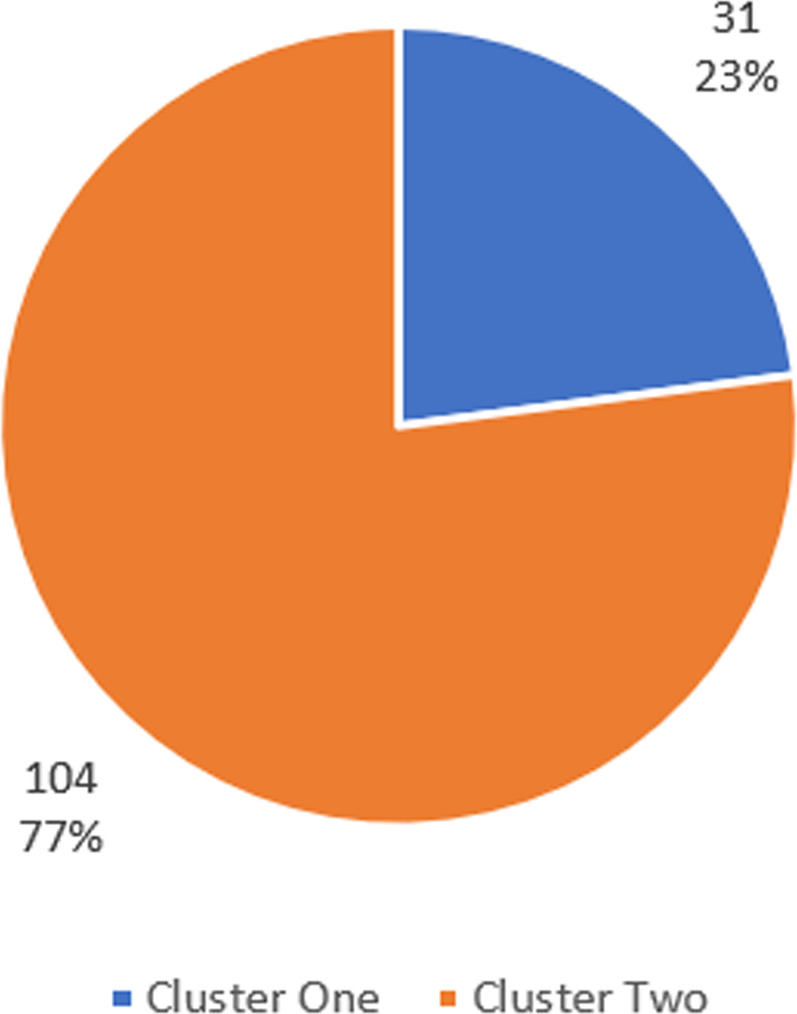
Number and percentage of KSA governorates in each cluster.

Before running the cluster analysis, data are prepared for clustering by transforming all the variables to *z*-scores to get rid of unit scale. Next the hierarchal cluster technique is invoked to find the best number of clusters. The last 10th rows of the agglomeration schedule table are presented in Table (A-4). Silhouette coefficients in addition to the scree diagram plot are employed to find the ideal number of clusters. As presented in Figs. 2 and 3 two is the optimal value for number of clusters. The *K*-means cluster method is applied with *K* equal to two. Figure 4 highlights the distribution of Saudi governorates over clusters. The first cluster, which accounts for 23% of KSA governorates, is characterized with relatively high number of infections in contrast to the second cluster that includes 77% of all KSA governorates.

**Table 4:** Cluster centers for *K*-mean cluster.

Variable	Cluster	
	1	2
*l.POPULATION*	1.31	−0.57
*l.INFECTED.CASES*	1.53	0.51
*l.HOUSES*	0.13	−0.04
*l.HOSPITALS*	1.06	−0.32
*l.NURSES*	1.52	−0.45
*l.DOCTORS*	1.52	−0.45
*l.BEDS*	1.54	−0.46
*l.MSCHOOLS*	1.29	−0.39
*l.FSCHOOLS*	1.30	−0.39
*l.MAV INTENSITY*	0.84	−0.39
*l.FAV INTENSITY*	0.88	−0.39
*l.INSURANCE*	−0.04	0.01

**Fig. 5:**
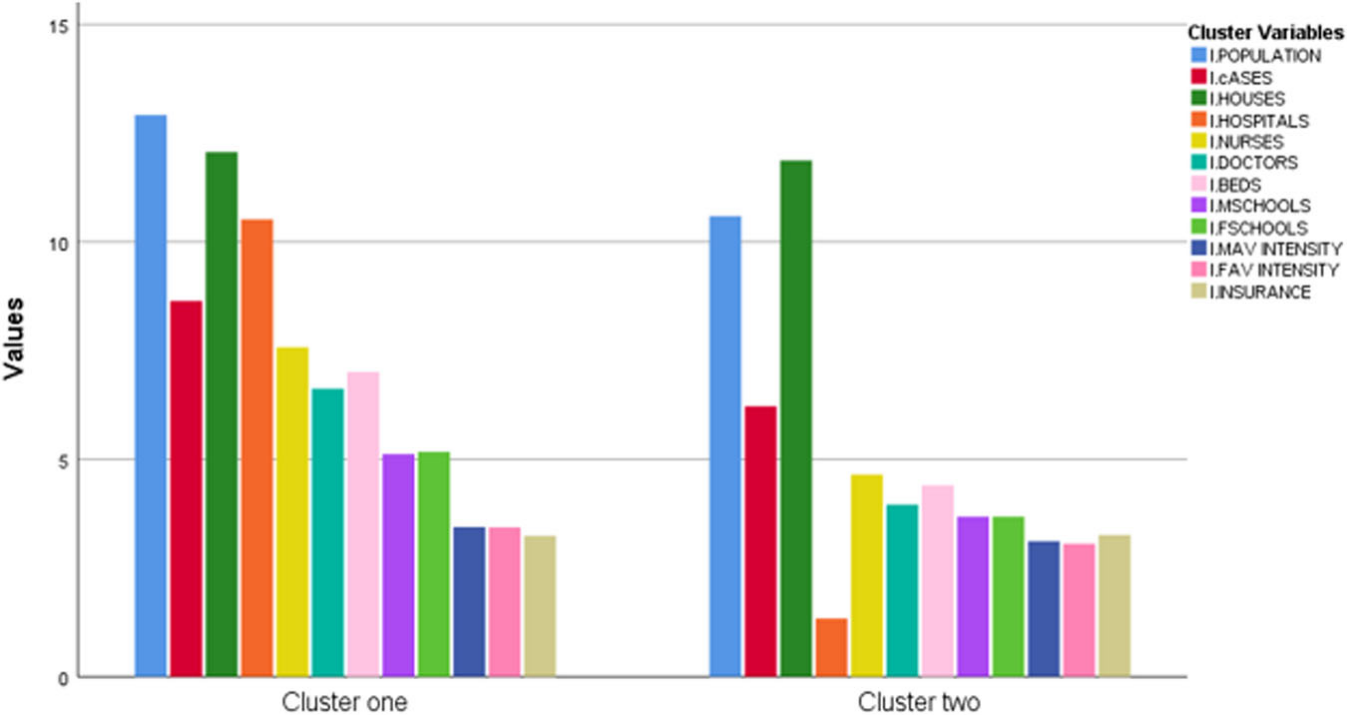
Means of the cluster variables for each cluster.

Figure 5 shows the means of each cluster while Table 4 shows cluster centers for all clusters. As presented, it is clear that governorates with higher number of infections are grouped with one cluster. In addition, these governorates have also higher values in other cluster variables except for *l.INSURANCE*. These findings coincide with the results obtained from principal regression model that link number of infections with higher values in model covariates and lower values in *l.INSURANCE*.

### Forecasting daily number of COVID-19 cases

In this part, the performance of SARIMA models, SES methods, MLP and LSTM networks are validated using the series of daily infected cases for each cluster constructed using *K*-mean cluster method in sub-section “Cluster analysis results”. For each cluster, the infected cases time series is divided to training set and test set such that training set represents the first 89% of the series leaving last 11% for test set. Whence all the methods under comparison are trained using data in the interval “March 30, 2020 to May 22, 2021” and comparison is accomplished using data in the interval “May 23, 2021 to July 11, 2021”.

Unfortunately, data in all clusters have many missing values as not all the governorates submit the number of infected cases every day in the study period. Dealing with missing values is crucial and mainly depends on the missingness mechanism that generates missing (Yaseen et al., 2016). If the missingness is missing completely at random (MCAR) or missing at random (MAR), traditional and classical imputation techniques can be used. Sophisticated and complicated methods must be entailed if the missingness type is missing not at random (MNAR) (Yaseen and Gad, 2020). Herein we do not have any reason to assume the missingness is MNAR, therefore we can impute all the missing values using linear interpolation which suits time series nature.

We prepare the data by imputing all missing values and then applying the logarithm transformation on the series of the two clusters to reduce heterogeneity. The next step is to set the predictors of the forecasting model. Since we have no time related independent variables, time is used as predictors, i.e., year, month, quarter, week of year and day are included as independent variables.

After setting up the model and preparing data for analysis, the compared methods are run based on the data of each cluster. For the SES technique and SARIMA models, the parameters of the models are set using SPSS expert modeler, which allows choosing the best parameters values according to the estimated forecast errors. Table 5 shows the estimated parameters of each model. For MLP networks, SPSS determined the best number of neutrons in the hidden layer, and number of epochs. A common rule of thumb for determining number of neutrons in the hidden layer is to choose a value between the number of inputs and number of outputs (Hornik, 1991). SPSS package set the number of neutrons in the hidden layer using the automatic structure as 4. Hyperbolic tangent activation function is selected for the hidden layer and identity activation function is used for the output layer. The MLP network applies batch training using scale conjugate gradient optimization algorithm with the default values of SPSS v.26 for training settings. For the LSTM network, a variety of values are attempted for the number of epochs, number of cells, and initial rates, determining that the best performance is accomplished with 600 epochs, and with four neutrons in the hidden layer, 0.3 for the initial rate, and 0.2 for the learning rate drop factor. Adam optimizer is enveloped as the back-propagation algorithm. Different alternatives for the previous settings are tested, yet no noticeable change is detected. Table 5 summarizes model settings for each of the comparison technique.

**Table 5:** Estimated parameters for the forecasted models.

Model	SARIMA	SES	MLP	LSTM
Cluster one	ARIMA(0,1,6)(0,0,1)	Winters’ Additive: alpha: 0.715, Beta: 0.04, Gamma: 0.007	Type of training: Batch, Optimization algorithm: Scaled conjugate gradient, Max Epoch: 500 *Input layer* Number of units: 5, rescaling of covariates: standardized. *Hidden layer* Number of hidden layers: 1, number of units: 4. Activation function: Hyperbolic tangent. *Output layer* Error Function: Sum of Squares	Activation function: TanH, Dropout factor: 0.20, Epochs: 300, Learn Rate Schedule: piecewise, Learn rate: 0.008, Learn Rate Drop Period: 125, Gradient Threshold: 1, Hidden layer :1, Cells in the Hidden layer:7, Solver: Adam, loss function: Mean square error
Cluster two	ARIMA(3,1,0)(1,0,1)	Holt: alpha: 0.49, Beta: 0.04		

After the training process is accomplished, fitted values are calculated for all techniques to validate the models’ accuracy. Testing the behavior of the different methods is undertaken according to three different criteria which are; mean square error (MSE), root mean square error (RMSE), and mean absolute deviation (MAD) that can be expressed as

$${\rm {MSE}} = \frac{1}{n}{\sum} {\left( {y - \widehat y} \right)^2}$$

$${\rm {RMSE}} = \sqrt {\frac{1}{n}{\sum} {\left( {y - \widehat y} \right)^2} }$$

and

$${\rm {MAD}} = \frac{1}{n}{\sum} {\left| {y - \widehat y} \right|}$$

where *y* and $\widehat y$ represent the actual and forecasted data for *n* time points.

Table 6 highlights the comparison metrics for all clusters. MLP achieves the lowest values in all comparison criteria for both clusters, followed by SARIMA models. In contrast, SES method has the largest forecasting error in both clusters. This indicates that the MLP network is the ideal method for predicting the future infected cases.

**Table 6:** Forecasting errors measures for the four compared methodsis.

● Arrows , , ,  represent the order of values within each row from the minimum to maximum.

The comparison between the different forecasting techniques clearly reveals that MLP is the most accurate model for the study clusters. This result coincides with the opinions reported in the literature over the superiority of neural networks over classical statistical models (see, for instance Elwasify, 2015; Hossain et al., 2017).

**Fig. 6:**
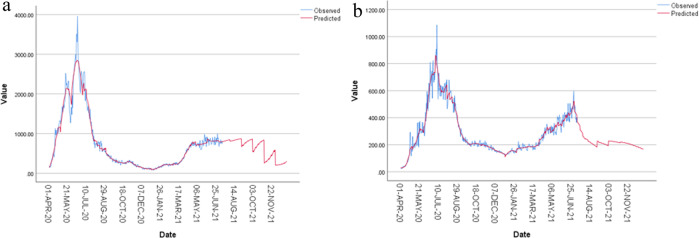
Forecasting the daily number of Covid infections in KSA governorates clusters. **a** Observed and expected number of daily Covid infections for cluster one. **b** Observed and expected number of daily Covid infections for cluster two.

Finally, MLP is used to forecast the future number of infections in both clusters till December 31, 2021. Figure 6a, b plot the observed and forecasted values for each cluster. It is noticed, in general, the daily number of infected cases will decrease in both clusters. This is logical in the shade of the adopted precautionary measures and vaccine campaigns the KSA holds all over the kingdom. For cluster two, it seems the rate of decrease is larger than cluster one’s decreasing rate. This is logical since cluster one has the governorates with the highest values in infections related factors that positively affect number of infections.

## Conclusion

This study addresses multiple research objectives. The first is to investigate the reasons behind the differences between KSA governorates, with respect to the cumulative number of COVID-19 cases. Results reveal that several factors explain the change in the cumulative number of COVID-19 cases. In addition to population size and density, the high number of schools and high classroom density inside these schools are associated with high number of infections. These variables contribute directly and indirectly to the increase in close contact, and the decrease in social distancing between citizens. In contrast, the number of employees does not have a significant effect, which sheds light on the validity of the precautionary measures inside workplaces, not including educational institutions. In addition, the study’s regression model proves that governorates with higher infections receive more medical care and resources, which suggests the adoption of efficient strategies to deal with the pandemic. Moreover, the second aim of this study is to classify the KSA governorates using the cumulative number of infections, and the significant predictors associated with it. Results reveal that all KSA governorates can be categorized into two groups. Clustering KSA governorates using number of Covid infections in addition to its determinants provides more information about which areas severely suffer from Covid pandemic and how to efficiently deal with it. Finally, different methods are adopted to predict the future number of daily infections for each cluster. Results highlight the superiority of MLP over all other comparing techniques. Fortunately, the forecasted data show a sharp decrease in the number of infections for cluster two, which includes most of KSA governorates. Overall, this study concludes that maintaining the current governmental strategies regarding COVID-19 is the best measure to keep the infections in the KSA to a minimum. Stricter strategies, such as decreasing classroom density in schools, may be needed for governorates with high number of schools and classroom density.

## Supplementary information

Appendix

Figure A-1

## Data Availability

All the study datasets are publicly published in KAPSARC data portal (ww.datasource.kapsarc.org) and Saudi General Authority for Statistics (www.stats.gov.sa).

## Footnotes

1 https://datasource.kapsarc.org/explore/dataset/saudi-arabia-coronavirus-disease-covid-19-situation.
