# Correction: Impact of social determinants on COVID-19 infections: a comprehensive study from Saudi Arabia governorates

**DOI:** 10.1057/s41599-022-01448-2

**Published:** 2022-12-20

**Authors:** Abdallah S. A. Yaseen

**Affiliations:** National Centre for Social and Criminological Research, Giza, Egypt

**Keywords:** Health humanities, Medical humanities

Correction to: *Humanities and Social Sciences Communications* 10.1057/s41599-022-01208-2, published online 07 October 2022.

In the original version of this article, incomplete versions of tables 2 and 6 were inadvertently published. These have now been corrected in full in the HTML and PDF versions of the paper.

Original table 2
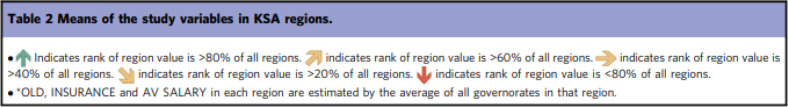


Amended Table 2

**Table 2:**
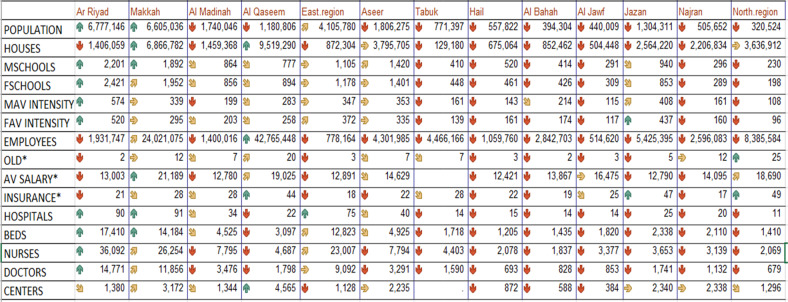
Means of the study variables in KSA regions.

indicates rank of region value is greater than 80% of all regions.  indicates rank of region value is greater than 60% of all regions.  indicates rank of region value is greater than 40% of all regions.  indicates rank of region value is greater than 20% of all regions.  indicates rank of region value is less than 80% of all regions.

*OLD, INSURANCE and AV SALARY in each region are estimated by the average of all governorates in that region.

Original table 6
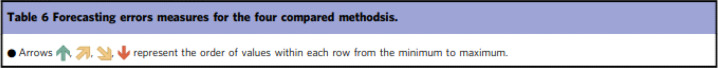


Amended Table 6

**Table 6:**
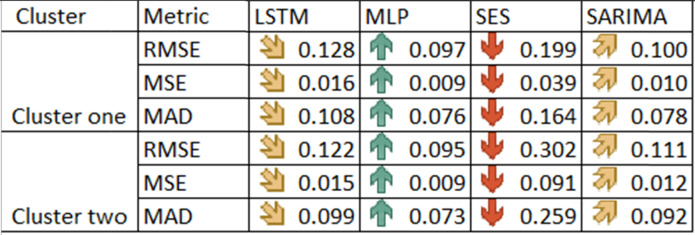
Forecasting errors measures for the four compared methods.

Arrows  represent the order of values within each row from the minimum to maximum.

