# Supplementary material for: Impact of social determinants on COVID-19 infections: a comprehensive study from Saudi Arabia governorates
Source: Humanit Soc Sci Commun. 2022 Oct 7;9(1):355. doi: 10.1057/s41599-022-01208-2 (PMC9540145; doi:10.1057/s41599-022-01208-2)
Supplement: Supplementary file 1 — Appendix [file 41599_2022_1208_MOESM1_ESM.docx]

**Appendix**

|  | l.population | l.0HOUSES | L.MSCHOOLS | L.FSCHOOLS | L.MAV INTENSITY | L.FAV INTENSITY | L.EMPLOYEES | OLD | L.INSURANCE | L.HOSPITALS | L.BEDS | L.NURSES | L.DOCTORS |
| --- | --- | --- | --- | --- | --- | --- | --- | --- | --- | --- | --- | --- | --- |
| $l.POPULATION$ | 1 |  |  |  |  |  |  |  |  |  |  |  |  |
| $l.HOUSES$ | 0.07 | 1 |  |  |  |  |  |  |  |  |  |  |  |
| $l.MSCHOOLS$ | .88^**^ | 0.12 | 1 |  |  |  |  |  |  |  |  |  |  |
| $l.FSCHOOLS$ | .89^**^ | 0.09 | .97^**^ | 1 |  |  |  |  |  |  |  |  |  |
| $l.MAV INTENSITY$ | .60^**^ | 0.02 | .32^**^ | .35^**^ | 1 |  |  |  |  |  |  |  |  |
| $l.FAV INTENSITY$ | .59^**^ | -0.01 | .30^**^ | .29^**^ | .79^**^ | 1 |  |  |  |  |  |  |  |
| $l.EMPLOYEES$ | 0.01 | .70^**^ | 0.07 | 0.06 | 0.04 | -0.09 | 1 |  |  |  |  |  |  |
| $OLD$ | -0.002 | .77^**^ | 0.02 | 0.01 | 0.04 | -0.06 | .89^**^ | 1 |  |  |  |  |  |
| $l.INSURANCE$ | 0.02 | .48^**^ | 0.05 | 0.04 | 0.12 | 0.13 | .66^**^ | .55^**^ | 1 |  |  |  |  |
| $l.HOSPITALS$ | .86^**^ | 0.02 | .72^**^ | .72^**^ | .49^**^ | .49^**^ | -0.07 | 0.01 | -0.12 | 1 |  |  |  |
| $l.BEDS$ | .90^**^ | 0.04 | .76^**^ | .77^**^ | .55^**^ | .53^**^ | -0.03 | 0.02 | -0.07 | .94^**^ | 1 |  |  |
| $l.NURSES$ | .90^**^ | 0.01 | .75^**^ | .76^**^ | .59^**^ | .58^**^ | -0.03 | 0.02 | -0.06 | .92^**^ | .96^**^ | 1 |  |
| $l.DOCTORS$ | .92^**^ | -0.01 | .77^**^ | .78^**^ | .60^**^ | .56^**^ | -0.04 | -0.01 | -0.07 | .92^**^ | .96^**^ | .97^**^ | 1 |

Table (A-1) Correlation matrix between predictors of the study regression model

- ** significant at 99% confidence interval
- * significant at 95% confidence interval

| Dimension | Eigenvalue | Condition Index |
| --- | --- | --- |
| 1 | 13.012 | 1.0 |
| 2 | 0.724 | 4.2 |
| 3 | 0.198 | 8.1 |
| 4 | 0.032 | 20.3 |
| 5 | 0.014 | 30.7 |
| 6 | 0.006 | 48.2 |
| 7 | 0.005 | 50.0 |
| 8 | 0.003 | 68.5 |
| 9 | 0.002 | 81.1 |
| 10 | 0.002 | 88.2 |
| 11 | 0.001 | 100.6 |
| 12 | 0.001 | 127.9 |
| 13 | 0.001 | 136.0 |
| 14 | 0.000 | 210.6 |

Table (A-2) Eigen values and conditional index of the study regression model

| Single global test |  | Value | P-value |  |
| --- | --- | --- | --- | --- |
|  | Global test Statistic | 1.914 | 0.752 | Assumptions acceptable. |
|  | Skewness | 0.487 | 0.485 | Assumptions acceptable. |
|  | Kurtosis | 0.001 | 0.976 | Assumptions acceptable. |
|  | Link function | 0.260 | 0.61 | Assumptions acceptable. |
|  | Heteroscedasticity | 1.17 | .28 | Assumptions acceptable. |
| Studentized Breusch-Pagan test |  | 21.89 | .06 | Assumptions acceptable. |
| Durbin-Watson test | D-W statistic | 2.01 | .66 | Assumptions acceptable. |

Table (A-3) Validating linear regression assumptions after applying Box-cox transformation

|  | | | | | | |
| --- | --- | --- | --- | --- | --- | --- |
| Stage | Cluster Combined | | Coefficients | Stage Cluster First Appears |  | Next Stage |
|  | Cluster 1 | Cluster 2 |  | Cluster 1 | Cluster 2 |  |
| 125 | 2 | 24 | 351.03 | 116 | 102 | 130 |
| 126 | 19 | 84 | 379.69 | 113 | 103 | 127 |
| 127 | 1 | 19 | 413.02 | 121 | 126 | 129 |
| 128 | 4 | 26 | 453.18 | 119 | 117 | 131 |
| 129 | 1 | 3 | 507.26 | 127 | 123 | 131 |
| 130 | 2 | 35 | 567.35 | 125 | 122 | 133 |
| 131 | 1 | 4 | 658.38 | 129 | 128 | 132 |
| 132 | 1 | 45 | 769.48 | 131 | 124 | 134 |
| 133 | 2 | 15 | 886.80 | 130 | 98 | 134 |
| 134 | 1 | 2 | 1521.70 | 132 | 133 | 0 |

Table (A-4) Last tenth rows of the agglomeration schedule in hierarchal cluster model

Figure (A-1) Scatter plot of studentized residuals and standardized predicted values before applying Box-Cox transformation on the study regression model
