# Supplementary figures and images for: Impact of social determinants on COVID-19 infections: a comprehensive study from Saudi Arabia governorates
Source: Humanit Soc Sci Commun. 2022 Oct 7;9(1):355. doi: 10.1057/s41599-022-01208-2 (PMC9540145; doi:10.1057/s41599-022-01208-2)

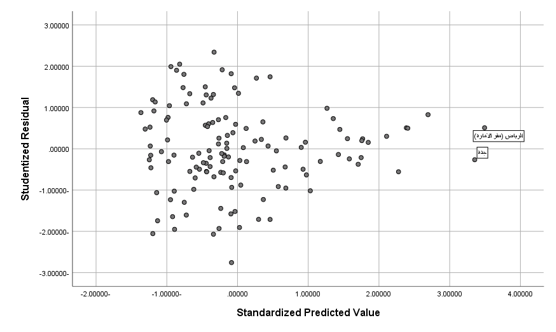

Supplement: Supplementary file 2 — Figure A-1 [file 41599_2022_1208_MOESM2_ESM.png]
